# Monitoring drug nanocarriers in human blood by near-infrared fluorescence correlation spectroscopy

**DOI:** 10.1038/s41467-018-07755-0

**Published:** 2018-12-13

**Authors:** Inka Negwer, Andreas Best, Meike Schinnerer, Olga Schäfer, Leon Capeloa, Manfred Wagner, Manfred Schmidt, Volker Mailänder, Mark Helm, Matthias Barz, Hans-Jürgen Butt, Kaloian Koynov

**Affiliations:** 1 Max Planck Institute for Polymer Research, Ackermannweg 10, 55128 Mainz, Germany; 2 Pharmaceutical Chemistry, Institute of Pharmacy and Biochemistry, Johannes Gutenberg University, Staudinger Weg 5, 55128 Mainz, Germany; 3 Institute of Physical Chemistry, Johannes Gutenberg University, Jakob Welder Weg 11, 55128 Mainz, Germany; 4 Institute of Organic Chemistry, Johannes Gutenberg University, Duesbergweg 10-14, 55128 Mainz, Germany; 5 Department of Dermatology, University Medical Center of the Johannes Gutenberg University, Langenbeckstr. 1, 55131 Mainz, Germany; 6 Earth-Life Science Institute, Tokyo Institute of Technology, Meguro, Tokyo, 152-8551 Japan

## Abstract

Nanocarrier-based drug delivery is a promising therapeutic approach that offers unique possibilities for the treatment of various diseases. However, inside the blood stream, nanocarriers’ properties may change significantly due to interactions with proteins, aggregation, decomposition or premature loss of cargo. Thus, a method for precise, in situ characterization of drug nanocarriers in blood is needed. Here we show how the fluorescence correlation spectroscopy that is a well-established method for measuring the size, loading efficiency and stability of drug nanocarriers in aqueous solutions can be used to directly characterize drug nanocarriers in flowing blood. As the blood is not transparent for visible light and densely crowded with cells, we label the nanocarriers or their cargo with near-infrared fluorescent dyes and fit the experimental autocorrelation functions with an analytical model accounting for the presence of blood cells. The developed methodology contributes towards quantitative understanding of the in vivo behavior of nanocarrier-based therapeutics.

## Abstract (web summary)

While nanocarrier-based drug delivery is a promising therapeutic approach, in situ characterization of drug nanocarriers in blood remains difficult. Here, the authors demonstrate how the fluorescence correlation spectroscopy can be used to directly characterize drug nanocarriers in flowing blood.

## Introduction

The site-specific delivery of small drug molecules, proteins or nucleic acids by nanometer sized carrier systems bears an enormous potential to improve diagnosis and therapy^1–3^. It offers unique possibilities for the treatment of various diseases ranging from cancer to viral or bacterial infections^4–8^. Nanocarriers (NCs) can protect the cargo from the environment during transport through the blood system and deliver it to target tissues and/or cells^9^. To increase accumulation at the target site, NCs should possess long circulation times in the blood stream without aggregation, decomposition, or substantial loss of their drug cargo. The high concentration of proteins, cells and other solutes in the blood, however, critically affects the NC’s integrity compared to aqueous buffer conditions at which the NCs are typically prepared and characterized^10^. This poses a challenge to the design and synthesis of efficient NCs. Thus, in spite of the exciting perspectives and the tremendous research efforts in the field, to date only a moderate number of NCs have entered clinical trials and only a few became first line therapies^11,12^.

For a directed development of efficient new NCs, it is essential to precisely monitor their properties such as size, drug loading, and stability in blood. However, none of the currently available experimental techniques allows such investigations. Here, we present a new methodology, based on fluorescence correlation spectroscopy (FCS), which allows direct monitoring of the size and loading efficiency of NCs in human blood at individual particle level and thus provides unique feedback for the design and optimization of efficient delivery systems.

Due to its very high sensitivity and selectivity^13^ the FCS technique has found numerous applications in fields ranging from cell biology^14,15^ to polymer, colloid, and interface science^16–20^. FCS is perfectly suited for studying the formation of NCs^21,22^, their drug loading^23,24^, stability^25–27^, interactions with plasma proteins^28–32^ and triggered release^33,34^. However, FCS has so far never been adapted to in situ blood measurements. The reason is that blood and biological tissues strongly absorb and scatter light from the visible part of the spectrum, where conventional FCS setups and common fluorescent labels operate.

Here, we show that this problem can be overcome by labeling NCs or their cargo with near-infrared (NIR) dyes that have excitation and emission wavelengths in the range 700–1100 nm. This range is within the so-called NIR window in biological tissue, where light has a maximum depth of penetration. Furthermore, a fully NIR-FCS setup, in which the wavelengths of the excitation laser and the detected fluorescence are within the NIR window, has to be used for the experiments.

## Results

### NIR-FCS experiments in aqueous solutions

**Fig. 1:**
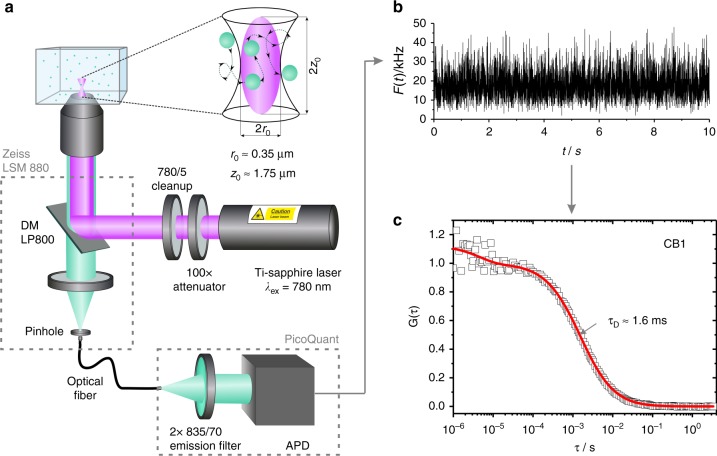
NIR-FCS experiments in aqueous solutions. **a** Schematic representation of the NIR-FCS setup. It is based on customized commercial equipment, modified so that both the excitation laser wavelength and the detected fluorescence are in the NIR spectral range. See Methods for details. **b** Fluorescence intensity time trace recorded for **CB1** and **c** the corresponding experimental autocorrelation curve (symbols). The red line represents a fit with Eq. (3)

Our NIR-FCS setup is schematically represented in Fig. 1a. It is based on commercial equipment that was properly customized in order to allow for NIR excitation and detection as described in the Methods. In brief, a microscope objective is used to tightly focus an excitation laser beam into a solution of the studied fluorescent species. The  emitted fluorescence light is collected by the same objective and after passing through a dichroic mirror, a confocal pinhole and an emission filter, it is delivered to a fast and sensitive photodetector (Fig. 1a). This arrangement results in the formation of a very small confocal observation volume *V*_obs_ of less than 1 μm^3^. Only fluorescent light originating from species that are in the observation volume can be detected. As the fluorescent species diffuse through the observation volume, they create fluctuations in the detected fluorescence intensity *F*(*t*) (Fig. 1b) that are recorded and evaluated by autocorrelation analysis. The obtained autocorrelation curve (Fig. 1c) is used to determine the mean residence time of the studied fluorescent species in the observation volume. From this time and the known size of the observation volume, one can calculate the diffusion coefficient and consequently the hydrodynamic radius of the studied fluorescent species.

However, the size of the confocal observation volume depends on the excitation laser wavelength and other specific characteristics of the FCS setup and is not known a priori. Therefore, it has to be first determined by performing a calibration with reference fluorescent species with known diffusion coefficient. In the wavelength range of visible light, common fluorescent dyes are used as reference, because there is a large library of literature data for their diffusion coefficients^35^. On the other hand, there are no literature reports for the diffusion coefficients of the recently developed NIR dyes. Thus, for calibration of our NIR-FCS setup we used cylindrical polymer brush macromolecules with a poly-l-lysine main chain and polysarcosine side chains^36^, labeled with IRDye®800CW-DBCO. Two cylindrical polymer brushes (**CB1** and **CB2)** were synthesized and labeled (see Methods for details). Both were large, stable, and of narrow size distribution and their diffusion coefficients in dilute solutions were reliably measured by multi-angle dynamic light scattering (Supplementary Figure 1). The diffusion coefficient of **CB1** in aqueous solutions (water or PBS) at 23 °C was determined as *D*_**CB1**, water_ = 20.4 µm² s^−1^ and that of **CB2** as *D*_**CB2**, water_ = 10.9 µm² s^−1^. By application of the Stokes–Einstein relation (Eq. (5) in the Methods) this translates into a hydrodynamic radii of *R*_H,**CB1**_ = 11.4 nm and *R*_H,**CB2**_ = 21.3 nm, respectively. Typical intensity time trace and the corresponding FCS autocorrelation curve recorded on the NIR-FCS setup for **CB1** diffusing in water are shown in Fig. 1b, c, respectively. The FCS autocorrelation curve measured for the **CB2** in water is presented in Supplementary Figure 7. The autocorrelation curves could be fitted well with the analytical model function for one type of freely diffusing species^13^ (Eq. (3) with *m* = 1 in the Methods) yielding the respective diffusion times of **CB1** and **CB2**. We performed three independent measurements for each of the cylindrical polymer brushes and averaged them to obtain respectively *τ*_*D*,**CB1**_ = 1.54 ± 0.08 ms and *τ*_*D*,**CB2**_ = 2.93 ± 0.03 ms. We used these values and the explicit relation (*τ*_*D*_ = *r*_0_²/(4*D*), Eq. (4) in the Methods) between the diffusion time, the size of *V*_obs_ and diffusion coefficient to calibrate our NIR-FCS setup observation volume and obtained a value of *r*_0_ = 0.36 ± 0.02 µm for its lateral radius. After this calibration, the setup could be used for measuring the diffusion coefficients and hydrodynamic radii of fluorescent species for which these values are not known. As a demonstration we studied two commercially available NIR dyes, namely Alexa Fluor^®^ 790 and IRDye®800CW-DBCO. The experimental FCS autocorrelation curves for these dyes diffusing in water are shown in Supplementary Figure 2. The corresponding fits (Eq. (3) with *m* = 1 in the Methods) yielded the diffusion times and thus (through Eq. (4) in the Methods) the diffusion coefficients of the commercial NIR dyes. We obtained values of 280 ± 10 µm² s^−1^ for Alexa Fluor^®^ 790 and 245 ± 15 µm² s^−1^ for IRDye®800CW-DBCO in water at 23 °C. The later value is very close to the 251 ± 10 µm² s^−1^ at 23 °C that we measured for the same IRDye®800CW-DBCO in independent pulsed field gradient nuclear magnetic resonance (NMR) experiments (see Supplementary Methods and Supplementary Figure 3) that further confirms the proper calibration of our NIR-FCS setup.

In addition to diffusion coefficient, FCS experiments measure also the fluorescence brightness (FB) of the studied species (see Methods). By comparing the FB of, e.g., individual dye molecules to the FB of a macromolecule or nanoparticle that are labeled or loaded with the same dye molecules one may estimate the labeling (loading) efficiency. For example, by comparing the FB of **CB1** to that of an individual IRDye®800CW-DBCO molecule we estimated an average labeling efficiency of 2.8 ± 0.7 dye molecules per cylindrical polymer brush, which is in accordance with the used molar equivalents of dye during the labeling procedure as described in the Methods.

### NIR-FCS experiments in human blood

The NIR fluorescent species (**CB1**) were dissolved in heparin-stabilized human blood. Blood, however, is a densely crowded medium with red blood cells occupying 40–45% of the volume. Therefore, one problem arises even in static blood: in order to observe unhindered Brownian diffusion of the fluorescent species, the FCS observation volume had to be positioned in a cell free spot (Supplementary Figures 4 and 5 and Supplementary Note 1). The NIR-FCS measurements in static blood therefore needed to be preceded by a time-consuming and tedious search for appropriate positions of the FCS observation volume and could provide only approximate qualitative information (Supplementary Note 1).

**Fig. 2:**
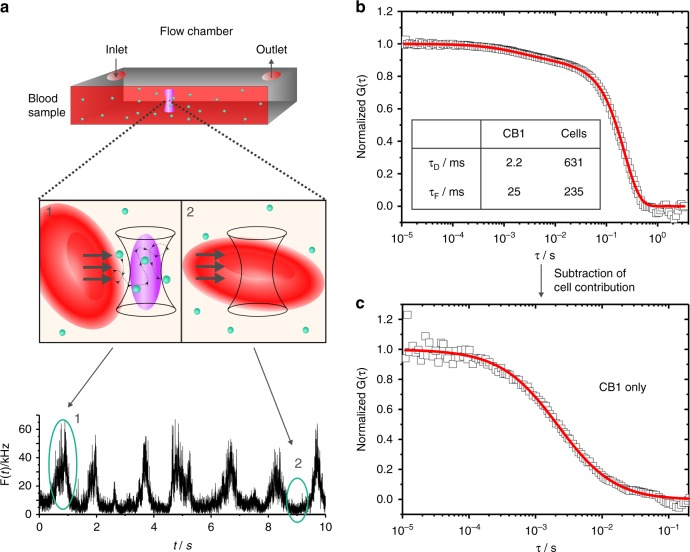
Overview of the NIR-FCS experiments and data analysis in flowing blood. **a** Monitoring NIR fluorescent species in flowing blood. Blood containing **CB1** (8 nM) was pumped through a flow channel at a defined velocity of 50 µL h^−1^. The FCS observation volume was consequently either free (schematics 1) or occupied (schematics 2) by a blood cell. Correspondingly, the fluorescence intensity time trace revealed high (1) and low (2) intensity time segments. **b** The experimental autocorrelation curve (squares) was fitted (line) with analytical model, Eq. (3), combining standard and inverse FCS thus taking into account contributions of fluorescent species and blood cells, respectively. **c** The information extracted from the fit in panel (**c**) was used to subtract the cells’ contribution and obtain an autocorrelation curve (squares) resembling that of a standard FCS experiment (Eq. ( 6)). A fit with Eq. (1) (line) yields diffusion properties of the fluorescent species

This problem was circumvented by introducing a directed movement to the blood. In flow, a dependence on measurement position is abrogated, as the occupation of *V*_obs_ by cells is only temporary. When measuring the fluorescence intensity in flowing blood containing fluorescent species, e.g., **CB1**, the intensity time trace alternated between two states (Fig. 2a): high intensity segments, occurring every 1–2 s, which were interrupted by low signal intervals. As schematically shown in Fig. 2a, the FCS observation volume was free of cells and accessible for the fluorescent species within high-intensity segments. In contrast, low intensity segments constituted times in which *V*_obs_ was occupied by a cell.

The experimental autocorrelation curve recorded under these conditions (Fig. 2b) shows two decays. The first one is at around few ms and was caused by the diffusion and flow of the fluorescent cylindrical polymer brushes **CB1** through the FCS observation volume. The second decay is at few hundred ms and was caused by the passage of blood cells. Therefore, in order to determine the diffusion of tracers two effects have to be considered: The presence of flow and the depletion of tracers in the presence of a blood cell. Below we discuss an analytical model that can be used to separate the two contributions.

In the presence of flow, the analytical model function for freely diffusing species (Eq. (3) in the Methods) has to be extended by an additional flow term^37^:

1 $${G_D\left( \tau \right) \ast G_F\left( \tau \right) = \left[ {1 + \frac{{f_T}}{{1 - f_T}}e^{ - \tau /\tau _T}} \right]\frac{1}{N}\mathop {\sum }\limits_{i = 1}^m \frac{{f_i}}{{\left[ {1 + \frac{\tau }{{\tau _{D,i}}}} \right]\sqrt {1 + \frac{\tau }{{S^2\tau _{D,i}}}} }} \ast e^{\left[ { - \frac{{\left( {\frac{\tau }{{\tau _F}}} \right)^2}}{{1 + \left( {\frac{\tau }{{\tau _{D,i}}}} \right)}}} \right]}{.}}$$

Here, *τ*_*F*_ denotes the flow residence time which is linked to the flow velocity *v* by *τ*_*F*_ = (*r*_*0*_ + *R*_*H*_)/*v*, where *R*_*H*_ is the hydrodynamic radius of the fluorescent species. Eq. (1) can be directly applied for representing the contribution of the fluorescent species to the experimental autocorrelation function.

Accounting for the depletion of tracers by blood cells, however, is not that trivial. In an earlier work, Wennmalm et al. have considered so-called inverse-FCS by performing FCS type of measurements in a strongly fluorescent medium containing unlabeled particles^38^. Under such conditions the diffusion of the particles through the FCS observation volume causes drops in the high fluorescence intensity background in a way similar to the drops caused by the passage of blood cells in our experiments (Fig. 2a). It was shown^38^ that the inverse-FCS autocorrelation curves recorded for one type of freely diffusing unlabeled particles can be well fitted with the classical analytical model function (Eq. (3) in the Methods) yielding correct values for the size of the particles. Here, we extended this approach to the combined diffusion and flow of the blood cells in the presence of the fluorescent species and represented the contribution of the blood cells with a term similar to Eq. (1). Thus, we fitted the experimental autocorrelation curve recorded in the flowing blood (Fig. 2b) with the following analytical function:

2 $$\begin{array}{l}G_{total}\left( \tau \right) = \left[ {1 + \frac{{f_T}}{{1 - f_T}}e^{ - \tau /\tau _T}} \right]\frac{1}{N}\left( {p_1 \ast G_{D1}\left( \tau \right) \ast G_{F1}\left( \tau \right) + p_2 \ast G_{D2}\left( \tau \right) \ast G_{F2}\left( \tau \right)} \right)\\ = \left[ {1 + \frac{{f_T}}{{1 - f_T}}e^{ - \tau /\tau _T}} \right]\frac{1}{N}\left( {\frac{{p_1}}{{\left[ {1 + \frac{\tau }{{\tau _{D1}}}} \right]\sqrt {1 + \frac{\tau }{{S^2\tau _{D1}}}} }} \ast e^{\left[ { - \frac{{\left( {\frac{\tau }{{\tau _{F1}}}} \right)^2}}{{1 + \left( {\frac{\tau }{{\tau _{D1}}}} \right)}}} \right]} + \frac{{p_2}}{{\left[ {1 + \frac{\tau }{{\tau _{D2}}}} \right]\sqrt {1 + \frac{\tau }{{S^2\tau _{D2}}}} }} \ast e^{\left[ { - \frac{{\left( {\frac{\tau }{{\tau _{F2}}}} \right)^2}}{{1 + \left( {\frac{\tau }{{\tau _{D2}}}} \right)}}} \right]}} \right)\end{array}{.}$$

*p*_1_ and *p*_2_ are the fractional contributions of tracers and cells, respectively. While a precise analytical derivation of Eq. (2) is outside of the scope of this paper, the combination of normal and inverse FCS is justified by the order of magnitude difference in the sizes and thus in the diffusion times of the fluorescent species and the blood cells (see Supplementary Note 3). In Eq. (2) each contribution contains its own diffusion and flow terms. Although blood cells and fluorescent tracers flow with the same velocity *v*, their tremendous size mismatch results in two different flow residence times *τ*_*F*1_ and *τ*_*F*2_. The size of the fluorescent tracers is much smaller than the lateral dimension of the FCS observation volume *r*_0_ and thus *τ*_*F*1_ = *r*_0_/*v*, whereas the average size of red blood cells (≈8 µm in diameter and ≈2 µm in thickness) exceeds significantly the dimensions of *r*_0_ and thus determines *τ*_*F*2_.

The experimental autocorrelation curve measured for **CB1** in the blood flow could be fitted well with Eq. (2) (Fig. 2b). The fit parameters (inset in Fig. 2b) confirmed that both diffusion and flow times of the cells were orders of magnitude larger than those of the fluorescent **CB1**. Thus, without loss of generality one can subtract the cell contribution (second part of Eq. (2)) and present the experimental autocorrelation curve in a way familiar for FCS data (see Eq. (6). Figure 2c shows an autocorrelation curve of **CB1**, devoid of cell effects. Using the diffusion time of the **CB1** resulting from the fit (Eq. (1)), τ_*D*1_ = 2.2 ms, and the value of the lateral dimension of the probing volume, *r*_0_ = 0.355 µm, we calculated the diffusion coefficient *D*_**CB1**, blood_ = 14.4 µm² s^−1^ of **CB1** in blood. The value is lower than the one measured for the same cylindrical polymer brush in water *D*_**CB1**, water_ = 20.4 µm² s^−1^, but basically identical to the value measured in undiluted human plasma *D*_**CB1**, plasma_ of 14.3 µm² s^−1^ (Supplementary Note 2 and Supplementary Figure 6). As human plasma constitutes basically the same medium as the blood, but without the blood cells, the identical values of *D*_**CB1**, blood_ and *D*_**CB1**, plasma_ justify further the use of Eq. (2) and confirm that the FCS measurements in the blood flow delivers accurate results, which are undisturbed by the high fraction of blood cells.

As discussed in details in the Supplementary Note 2 the diffusion slowdown of **CB1** (and **CB2**) in plasma and blood with respect to water could be attributed solely to the higher viscosity of the plasma. Two further possible causes were ruled out. In short, neither an increase in *V*_obs_ caused by a refractive index mismatch in plasma (Supplementary Figure 6) nor a size increase of **CB1** or **CB2** due to the adsorption of proteins took place. We, therefore, used the ratio *D*_**CB1**,water_/*D*_**CB2**,plasma_ = *D*_**CB2**,water_/*D*_**CB1**, plasma_ = 1.43 (Supplementary Note 2) to determine the value of 1.34 mPa × s (at 23 °C) for the effective viscosity experienced by the cylindrical polymer brushes in plasma and blood. This value is only slightly lower than that of the macroscopic viscosity of the plasma *η*_plasma, 22 °C_ = 1.48 mPa × s as measured by rolling ball viscosimetry.

### Sensitivity of the NIR-FCS in flowing blood

**Fig. 3:**
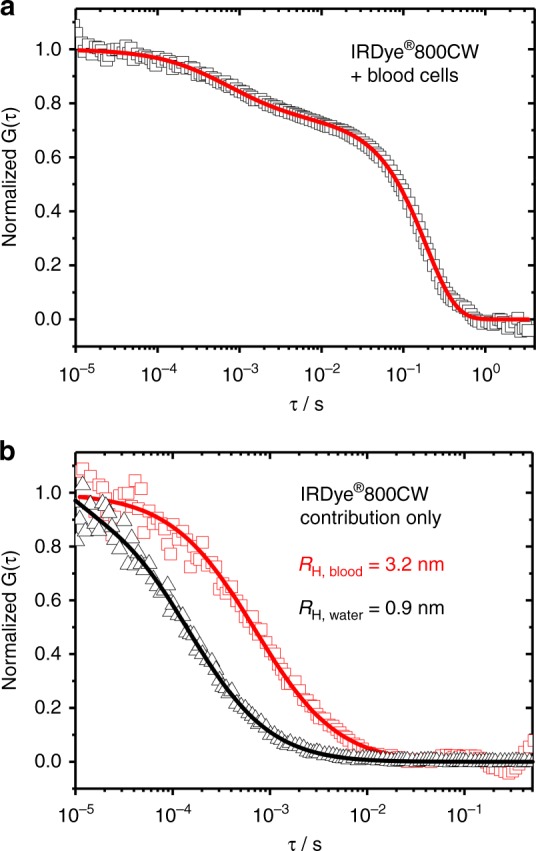
NIR-FCS measurement of IRDye®CW800-DBCO in flowing blood. **a** Autocorrelation curve fitted with Eq. (2) comprising contributions from fluorescent dyes and blood cells. **b** Contribution from the blood cells was subtracted (Eq. (6)) from the autocorrelation curve of IRDye®CW800-DBCO in blood (red). For comparison, the autocorrelation curve of IRDye®CW800-DBCO in water (black) is also shown

The cylindrical polymer brush **CB1** is relatively large (*R*_*H*_ = 11.4 nm) and labeled with in average three NIR dyes per particle. Therefore, we evaluated the sensitivity of NIR-FCS using single IRDye®800CW-DBCO molecules dissolved at 10 nM concentration in blood. A typical autocorrelation curve and the corresponding fit with Eq. (2) are shown in Fig. 3a. The fit yielded the value of the diffusion coefficient of IRDye®800CW in blood *D*_**IRDye®CW800**, blood_ ≈50 µm² s^−1^. For comparison we measured the diffusion coefficient of the same dye in undiluted human plasma (Supplementary Figure 8a) and obtained a value *D*_**IRDye®CW800**, plasma_ ≈38.1 µm² s^−1^. The similar values measured in blood and plasma confirmed further the validity of the used combination of FCS and inverse FCS (Eq. (2)) in the blood studies. After accounting for the effective plasma viscosity the diffusion coefficients measured in blood and plasma translate to hydrodynamic radii of 3.2 and 4.3 nm, respectively. These values are significantly larger than the hydrodynamic radius of IRDye®800CW-DBCO (≈0.9 nm) but very similar to the hydrodynamic radius of the human serum albumin (≈3.5 nm)^39^, indicating that IRDye®800CW probably binds to human serum albumin. Thus, we studied the diffusion of IRDye®800CW-DBCO in pure human serum albumin solutions and found a similar radius of 4.0 nm (Supplementary Figure 8b). These results are in line with the reported one-to-one complex formation of NIR dyes with human serum albumin^40–42^. Thus, we conclude that the NIR-FCS setup has sufficient sensitivity to monitor individual dye molecules in flowing blood and even their interactions with plasma proteins.

### Loading stability of drug NCs in blood

**Fig. 4:**
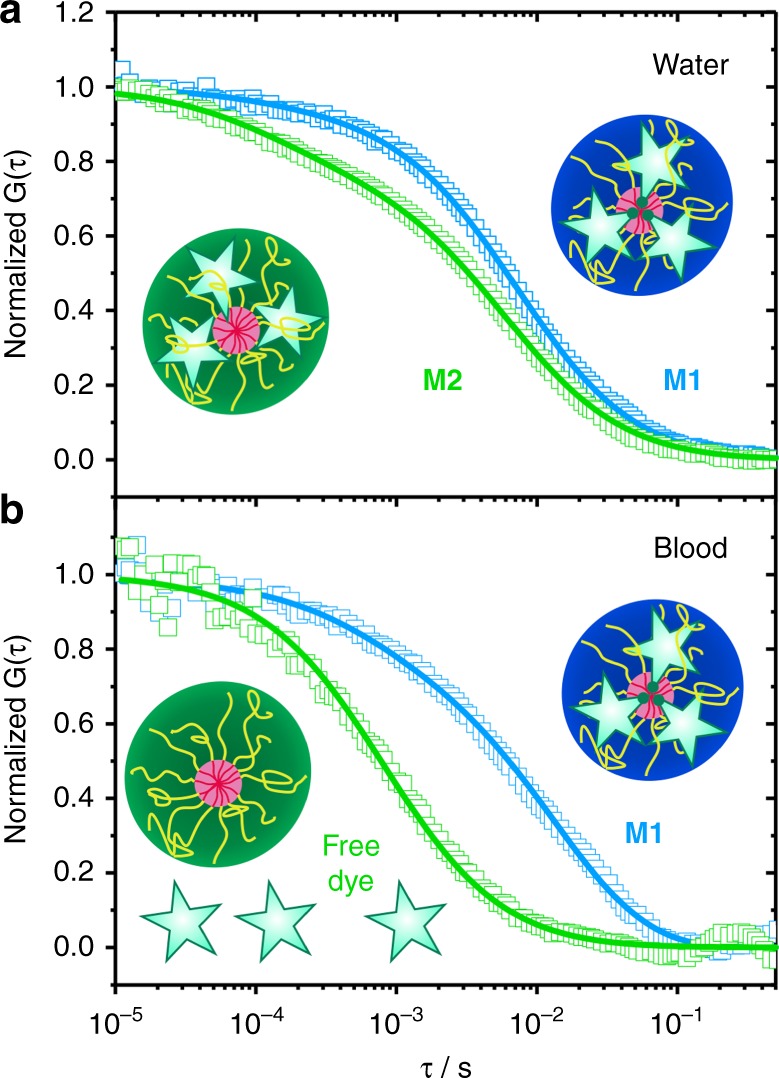
NIR-FCS studies of the loading stability of core-crosslinked micelle nanocarriers in blood. Normalized autocorrelation curves (symbols) and the corresponding fits (lines) are shown for core-crosslinked micelles that were either covalently (**M1**, blue color) or noncovalently (**M2**, green color) loaded with IRDye®800CW. **a** Measurements in water. The dye is mainly loaded to the core-crosslinked micelles and only a small fraction of free dye was detected for both systems. **b** Measurements in the blood flow (velocity of 50 µL h^−1^) upon incubation with blood for 30 h (at 4 °C). The dye is fully released from **M2**, but still loaded to **M1**

To demonstrate how the NIR-FCS method can be used to monitor the behavior of drug loaded NCs in blood, we investigated the loading stability of core-crosslinked micelles based on polypept(o)ides^43,44^. The core-crosslinked micelles were loaded with IRDye®800CW, which was used as a model for a small drug molecule. IRDye®800CW was either covalently (**M1**) or noncovalently (**M2**) attached to the NCs (see Methods). A first NIR-FCS characterization in water revealed hydrodynamic radii for **M1** and **M2** of 51 and 44 nm, respectively (Fig. 4a). These values were in accordance with the multi-angle dynamic light scattering data obtained for the unloaded core-crosslinked micelles (*R*_*H*, **M**_ = 45 nm, see Supplementary Table 2) and indicated that the IRDye®800CW molecules were indeed loaded to the core-crosslinked micelles. Nevertheless, the autocorrelation curves had to be fitted with two components (*m* = 2 in Eq. (3)), which indicated that in addition to the loaded core-crosslinked micelles a second type of fluorescent species were present in the solutions. These species had a hydrodynamic radius *R*_*H*_ ≈0.9 nm and thus were identified as free dye. The two component fits yielded also the apparent fractions (*f*_1_ and *f*_2_ in Eq. (3)) of the loaded micelles and the free dye in each case. We obtained free dye fractions of 5% for **M1** and 25% for **M2**. After 30 h of incubation in blood, the micelles were subjected to NIR-FCS measurements in flowing blood. The processed autocorrelation curves (Eq. (6)) devoid of cell contributions are shown together with the corresponding fits with Eq. (1) in Fig. 4b.

As in water, two components fit of the autocorrelation curve measured for **M1** revealed the presence of two types of fluorescent species with different hydrodynamic radii of 55 and 2.9 nm. The larger species with fraction *f*_1_ ≈0.8, were the dye loaded core-crosslinked micelles and the smaller one with fraction *f*_2_ ≈0.2 were the free dye molecules that have formed one-to-one complexes with plasma proteins. Thus, the fraction of free dye had increased from roughly 5% in water to about 20% in blood. This increase could be a result of a partial degradation of the peptide-dye bond and/or the presence of a small fraction of dyes that were non-covalently attached and thus dissociated from the core-crosslinked micelles in blood. The dominant fraction of the dye cargo, however, was still loaded on the core-crosslinked micelles which were intact and did not change their size, e.g., due to decomposition or aggregation even after 30 h in blood.

The autocorrelation curve for **M2** in blood (Fig. 4b) can be fitted well with a single component fit (Eq. (1) with *m* = 1) revealing that only one type of fluorescent species with *R*_*H*_ ≈3.4 nm are present in the blood. This indicates a complete loss of the noncovalently attached dye cargo from the NCs in blood, which is in line with observations on the loss of encapsulated dexamethasone or paclitaxel from core-crosslinked polymeric micelles in vivo^45–47^. The reason that the non-covalently attached IRDye®800CW-DBCO stays loaded in the core-crosslinked micelles **M2** in water, but is leaking from them in blood is the presence of proteins in blood. Even hydrophobic dyes or drugs have a limited solubility in water allowing transfer to hydrophobic binding pockets in proteins, leading to a loss of dye or drug from the core of the micelle.

It is particularly interesting to use the developed NIR-FCS method for measuring the kinetic of the leakage of encapsulated dyes or fluorescent drugs. Thus, as prove of principle, we performed experiments with **M2** incubated in blood for different time intervals. However, as shown in Supplementary Figure 9, it has turned out that the dyes are completely released from **M2** already after the first incubation time interval of 30 min. This fast release prevents detailed kinetic study for **M2**.

## Discussion

We showed how a slightly modified commercial FCS setup that employs NIR excitation and emission can be used to monitor the size and drug loading of NCs in blood. With its sensitivity down to the level of single dye molecules, the method is suitable to study the stability, premature release, interaction with blood components or aggregation of NCs in blood. Although our focus was on monitoring of NCs loading stability, the method can be also applied to study the size or detect possible interactions of other exogenously introduced NIR labeled species, such as proteins, RNA, or DNA^48,49^.

Furthermore, the formation of a protein corona on the NCs can be investigated by NIR-FCS. While FCS in the visible range has been successfully applied to determine kinetic parameters on the binding affinities of proteins as well as the thickness of the adsorbed protein layer(s) in blood serum or plasma^28,50,51^, measurements directly in blood have never been reported. Only very recently, Carril et al. demonstrated that ^19^F diffusion-ordered NMR spectroscopy can be used to study the formation of a protein corona on model ^19^F-labeled gold nanoparticles directly in a blood sample^52^. While this method elegantly bypasses the need for optical detection, we believe that the very high sensitivity of NIR-FCS that can detect even one-to-one complex formation between a dye molecule and a plasma protein will help to obtain complementary information and improve our understanding of the interaction between NCs and blood components. Clearly, the method has also intrinsic limitations and similar to other methods based on monitoring only the size increase of the NCs upon formation of protein corona it cannot provide direct information on the type of proteins that are forming a complex corona.

The NIR-FCS method described above offers the possibility of performing ex vivo kinetic measurements by drawing blood samples at regular intervals and determining the stability and blood circulation half-life of fluorescently labeled NCs. Here, major advantage of the NIR-FCS in comparison to conventional methods is that it does not require separation of solid (cells) and liquid (plasma) components of the blood. Such separation is commonly done by centrifugation that can affect the NCs properties and integrity and provide inaccurate or even misleading results. Furthermore, conventional methods that measure the average fluorescence or absorbance only, cannot distinguish between encapsulated and released drug molecules. In contrast, NIR-FCS provides information not only on the circulation time by measuring the average fluorescent intensity and estimating the concentration of the fluorescent species in blood, but also on the loading stability of the NCs by distinguish between free drug molecules (fluorescent or labeled) and loaded NCs, due to their size difference. The method can even estimate how many drug molecules are loaded on a NC by comparing the FB (see Methods) of a loaded NC to that of a single drug molecule^16^. However, the accuracy of such estimation may be compromised if the used fluorescent molecules exhibit self-quenching at higher degree of loading e.g., due to aggregation^53,54^. Still, even in cases of very strong fluorescence quenching, FCS can monitor the free drug molecules and follow the increase of their concentration upon release from the NCs.

In conclusion, we expect that the presented methodology for NIR-FCS studies in flowing blood, will make important contributions to the quantitative understanding of the in vivo behavior of NCs and thus to the development of more efficient nanocarrier-based therapeutics.

## Methods

### NIR-FCS set-up

Our experimental setup (Fig. 1a) is based on a customized commercial confocal microscope (LSM 880, Carl Zeiss, Jena, Germany). Fluorescence is excited by a Ti:Sa laser (Mai Tai, Newport) tunable in the range 780–920 nm and delivering ≈100 fs pulses at 80 MHz repetition rate with ≈1 W average power. Although the use of such powerful femtosecond laser for linear excitation in NIR-FCS experiments is not necessary we choose to couple the Mai Tai laser to our confocal microscope for two pragmatic reasons. First, it allows performing two-photon excitation and second harmonic generation studies on the same microscope. Second, it allows convenient tuning of the excitation wavelength. In the experiments described in this paper, the laser was operated at 780 nm and strongly (≈10,000 times) attenuated using neutral density filters and an acousto-optical attenuator. The excitation laser beam was passed through a cleanup filter (to narrow down the broad excitation line) and a dichroic mirror and focused into the sample by a high numerical aperture water immersion objective (C-Apochromat 40×/1.2 W, Carl Zeiss, Jena, Germany). In most NIR-FCS experiments the average laser power after the objective was kept below 6 µW to avoid saturation effects (Supplementary Figure 2c). The fluorescence was collected with the same objective and after passing through the dichroic mirror and a confocal pinhole (54 µm) delivered to an avalanche photodiode detector (Excelitas, Waltham, MA, USA), integrated in a PicoQuant FLIM and FCS upgrade kit and fiber coupled to the microscope.

### NIR-FCS static measurements

FCS measurements in static conditions (water, plasma, and blood) were performed in an eight-well polystyrene chambered cover glass (Nunc, Thermo Fisher Scientific, Waltham, MA, USA) at 23 °C. Measurements were performed at 30–60 µm (water), 30 µm (plasma), and 10 µm (blood) depth of penetration. The total measurement time per sample ranged between 120 and 900 s and consisted of time segments (repetitions) of 10–30 s each. The time dependent fluctuations of the fluorescence intensity *F*(*t*) caused by the diffusion of the fluorescent species through the confocal observation volume *V*_*obs*_ were recorded and analyzed by an autocorrelation function *G*(*τ*) = 〈*δF*(*t*)∙*δF*(*t* + *τ*)〉/〈*F*(*t*)〉^2^. Fitting of these experimental autocorrelation functions was performed with ZEN software (Carl Zeiss, Jena, Germany). Hereby, repetitions which revealed the occasional presence of large aggregates were deselected prior to the fitting.

The analytical expression for the autocorrelation function of an ensemble of *m* types of fluorescent species has the following form^13^:

3 $$G_D\left( \tau \right) = \left[ {1 + \frac{{f_T}}{{1 - f_T}}e^{ - \tau /\tau _T}} \right]\frac{1}{N}\mathop {\sum }\limits_{i = 1}^m \frac{{f_i}}{{\left[ {1 + \frac{\tau }{{\tau _{D,i}}}} \right]\sqrt {1 + \frac{\tau }{{S^2\tau _{D,i}}}} }}{.}$$

Here, *N* is the mean number of fluorescent species in the observation volume *V*_*obs*_ and *f*_*T*_ and *τ*_*T*_ are the fraction and the decay time of the triplet state. *S* *=* *z*_0_/*r*_0_ (≈5 for our setup) is the so-called structure parameter, with *z*_0_ and *r*_0_ representing the axial and lateral dimensions of *V*_*obs*_. *τ*_*D,i*_ is the average diffusion time which the fluorescent species *i* (1 ≤ *i* ≤ *m*) require to cross through *V*_*obs*_. The respective fraction of species *i* is given by *f*_*i*_. Note that *f*_*i*_ scales with the square root of the FB of species *i*. Therefore, if two or more types of fluorescent tracers show different brightness, respective *f*_*i*_ are not absolute fractions (termed here apparent fractions). The diffusion coefficients of the species *D*_*i*_ are related to the respective diffusion times *τ*_*Di*_ and the radial dimension *r*_0_ of *V*_*obs*_ by:

4 $$D_i = r_0^2/\left( {4\tau _{Di}} \right){.}$$

By inserting *D*_*i*_ into Stokes–Einstein Eq. (5), the hydrodynamic radius of the respective fluorescent species can be calculated. Here, *k*_*B*_ is Boltzmann’s constant, *T* is the temperature and *η* is the viscosity of the solvent.

5 $$R_{H,i} = \frac{{k_BT}}{{6\pi \eta D_i}}{.}$$

Furthermore, if only one type of fluorescent species are present in the studied solution (*m* = 1 in Eq. (3)) FCS yields also the FB of these species defined as the ratio between the detected average fluorescent intensity and the mean number of fluorescent species in the observation volume, FB = 〈*F*(*t*)〉/*N*.

### NIR-FCS flow measurements

Flow was generated with a syringe pump (kdScientific, Holliston, MA, USA). The syringe was connected by a tube to a microchannel consisting of a sticky-slide I luer and a glass coverslip (both from Ibidi, Martinsried, Germany). The dimensions of the channel were 0.1 × 5 × 48.2 mm in height, width and length. In order to calibrate the system, autocorrelation curves for **CB1** and IRDye®800CW dibenzocyclooctyne (LI-COR Biotechnology, Lincoln, NE, USA) were recorded in the same channels, also in the absence of flow.

For blood flow measurements, 3 mL of heparin-treated human blood containing fluorescent probes with nanomolar concentrations were loaded into the syringe and a flow rate of 50 µL h^−1^ was applied. At significantly higher flow rates, the time intervals in which the FCS observation volume was free of blood cells and thus accessible for the fluorescent NCs (high intensity intervals in Fig. 2a) became too short for recording accurate statistic. Flow rates of less than 50 µL h^−1^ did not change the obtained data. *V*_*obs*_ was positioned at 10 µm depth of penetration and fluorescence signal was detected for 300 s in repetitions of 10 s each. The fitting of the experimental autocorrelation curves was performed with OriginPro 9.1 (OriginLab, Northampton, MA, USA) using the analytical expression for two species *τ*_*D*1_ and *τ*_*D*2_ with their independent flow residence times *τ*_*F*1_ and *τ*_*F*2_ (Eq. 2). For simplicity and for reducing the number of fitting parameters the triplet term was neglected (*f*_*T*_ was fixed to 0 is Eq. (2)) and the autocorrelation curves were fitted only for lag times *τ* > 10 µs. Such approach is quite common when fitting FCS curves and is justified by the fact that the triplet time *τ*_*T*_ of the fluorescent dyes is in the order of few µs only and thus does not affect the autocorrelation curves at lag times longer than 10 µs. Our experiments in water showed that at the used excitation intensities the triplet time of IRDye®800CW-DBCO was *τ*_*T*_ ≈1.3 µs and its fraction *f*_*T*_ ≈0.28. Moreover, in the presence of multiple dyes per particle as for **CB1**, **CB2**, **M1**, and **M2** the effect of triplet is reduced even further. In order to subtract the cell contribution from the experimental autocorrelation curves and present them in a common for FCS form, the following mathematical operation was performed:

6 $$\begin{array}{*{20}{l}} {G_{\mathrm{processed}}\left( \tau \right)} \hfill & = \hfill & {\frac{{G_{\mathrm{total}}\left( \tau \right) \ast N - \left( {p_2 \ast G_{D2}\left( \tau \right) \ast G_{F2}\left( \tau \right)} \right)}}{{p_1}}} \hfill \\ {} \hfill & = \hfill & {\frac{{G_{\mathrm{total}}\left( \tau \right) \ast N - \frac{{p_2}}{{\left[ {1 + \frac{\tau }{{\tau _{D2}}}} \right]\sqrt {1 + \frac{\tau }{{S^2\tau _{D2}}}} }} \ast e^{\left[ { - \frac{{\left( {\frac{\tau }{{\tau _{F2}}}} \right)^2}}{{1 + \left( {\frac{\tau }{{\tau _{D2}}}} \right)}}} \right]}}}{{p_1}}} \hfill \end{array}{.}$$

The values for *p*_1_, *p*_2_, *τ*_*D*2_, and *τ*_*F*2_ used in Eq. (6) were derived from the fits of the original experimental autocorrelation curve with Eq. (2) (main text). Even when two fluorescent species were present in the studied system (e.g., loaded micelles **M1** and released dye) only one component with fraction *p*_1_, was used in Eqs. (2) and (6) to account for their combined contribution. The reason is that the difference between the sizes of these fluorescent species is significantly smaller than the difference to the very large blood cells and thus their individual contributions cannot be precisely determined at this stage. Thus the diffusion times and the fractions of these fluorescent species were determined at the next stage, by fitting the autocorrelation curve devoid of cell contributions with Eq. (1) using two components (*m* = 2 in Eq. (1)).

### Biological material

Plasma and blood were collected and handled according to the regulations and the votum of the ethics committee of the Landesärztekammer Rheinland-Pfalz. Human blood plasma was received from ten healthy donors in the Mainz University Medical Center. After addition of sodium citrate the donated blood was separated by centrifugation. The plasma was pooled and aliquots were stored at −80 °C. After thawing, the plasma was centrifuged at 20,000*g* for 30 min to remove any residual protein precipitates. The protein concentration was estimated to lie between 65 and 70 g L^−1^.

For FCS measurements in human blood, a male healthy donor volunteered to donate blood. The blood was collected in a heparin-coated tube (Sarstedt, Nümbrecht, Germany) to prevent clotting and was either immediately used or stored at 4 °C for up to 1 day.

### Synthesis and labeling of the cylindrical polymer brushes

Cylindrical polypept(o)ide brushes with polylysine backbone and polysarcosine side chains were synthesized according to Hörtz et al.^36^. The small cylindrical polymer brush (**CB1**) and large cylindrical polymer brush (**CB2**) differed in the repetition units of the polylysine backbone (**CB1** = 102 and **CB2** = 258 repetitive lysine units). Both cylindrical polymer brushes were labeled with IRDye®800CW DBCO. In case of **CB1**, 6 mg (33 nmol) of cylindrical polymer brushes were dissolved in 600 µL PBS and 18 µL (90 nmol) that is 2.7 equivalents IRDye®800CW DBCO in DMSO were added. For **CB2**, 42 mg (52 nmol) were dissolved in 2 mL PBS and 28 µL (140 nmol) that is 2.7 equivalents IRDye®800CW-DBCO in DMSO were added. After incubation for 24 h at 4 °C under light exclusion, the reaction mixture was purified by Amicon Ultra Centrifugal Filter Devices (15 mL, 50 kDa, 4000* g*, 10 times). Detailed characterization data can be found in Supplementary Table 1.

### Preparation and labeling of the core-crosslinked micelles

A poly(sarcosine)_203_-*b*-poly(S-ethylsulfonyl)cysteine)_11_ block copolymer^43^ was dissolved in dimethyl-acetamide (DMAc) with 1 M thiourea at a concentration of 7.5 g L^−1^ for 1.5 h to prevent β-sheet formation of the polycysteine segment. For self-assembly, 1 mM acetate buffer with 10 mM thiourea (pH = 4.7) was added to adjust the concentration to 6 g L^−1^. The solution was left to equilibrate for 3 h and then dialyzed against 1 mM acetate buffer. The cross-linker hexanedithiol was added as a DMAc solution to the micelle solution in 1 mM acetate buffer with 10 mM thiourea with SH-groups equimolar to the number of cysteines. The reaction mixture was shaken and allowed to stand for 18 h. Subsequently, the solution was dialyzed against water, filtered via GHP200 syringe filter, and purified by repetitive spin filtration (MWCO 100 kDa) and dilution steps. After the preparation steps described above, the particle solution was divided for covalent and noncovalent labeling with an equimolar amount of fluorophore in DMF (5 mM)^44^. The labeling reactions were carried out at room temperature overnight followed by purification. For covalent labeling, 10-fold concentrated PBS was added to adjust the pH to 8 and followed by 14.1 μL IRDye®800CW succinimidyl ester (LI-COR Biotechnology) solution. Purification was performed by repetitive spin filtration (MWCO 100 kDa) diluting at least ten times with EtOH/H_2_O followed by ten times H_2_O. For noncovalent labeling, 14.1 μL IRDye®800CW-DBCO solution were added to the aqueous particle solution followed by purification using column chromatography (Sephadex LH 20 in H_2_O).

### Multi-angle DLS

The cylindrical polymer brushes and the unlabeled core-crosslinked micelles were analyzed by multi-angle DLS. For the measurements, cylindrical quartz cuvettes (Hellma, Mühlheim, Germany) were cleaned with dust-free distilled acetone and transferred to a dust free flow box. Solutions were filtered into the cuvettes through Pall GHP filters, 0.2 μm pore size. DLS measurements were performed by the following instrument at 20 °C. The apparatus consists of a Uniphase He/Ne Laser (22.5 mW output power at *λ* = 632.8 nm), an ALV/SP125 goniometer with an ALV 5000/E/PCI correlator and an ALV/High QEAPD Avalanche photodiode detector. The correlation functions of the particles were fitted using a sum of two exponentials. The *z*-average diffusion coefficient *D*_*z*_ was calculated by extrapolating *D*_*app*_ for q = 0. By formal application of Stokes law, the inverse *z*-average hydrodynamic radius is *R*_*h*_ = 〈*R*_*h*_^−1^〉_*z*_^−1^.

### Rolling-ball viscosimetry

The viscosity of blood plasma was measured using a LOVIS 2000 M Microviscosimeter (Anton Paar GmbH).

## Supplementary Information

Supplementary Information

## Data Availability

The authors declare that the data supporting the findings of this study are available within the paper and its Supplementary Information files or from the corresponding author on reasonable request.

## References

[1] Mitchell MJ, Jain RK, Langer R (2017). Engineering and physical sciences in oncology: challenges and opportunities. Nat. Rev. Cancer.

[2] Pelaz B (2017). Diverse applications of nanomedicine. ACS Nano.

[3] Blanco E, Shen H, Ferrari M (2015). Principles of nanoparticle design for overcoming biological barriers to drug delivery. Nat. Biotechnol..

[4] Grabbe S, Landfester K, Schuppan D, Barz M, Zentel R (2016). Nanoparticles and the immune system: challenges and opportunities. Nanomedicine.

[5] Irvine DJ (2016). Materializing the future of vaccines and immunotherapy. Nat. Rev. Mater..

[6] Shi J, Kantoff PW, Wooster R, Farokhzad OC (2017). Cancer nanomedicine: progress, challenges and opportunities. Nat. Rev. Cancer.

[7] Singh L, Kruger HG, Maguire GEM, Govender T, Parboosing R (2017). The role of nanotechnology in the treatment of viral infections. Ther. Adv. Infect. Dis..

[8] Fenaroli F (2018). Enhanced permeability and retention-like extravasation of nanoparticles from the vasculature into tuberculosis granulomas in zebrafish and mouse models. ACS Nano.

[9] Lammers T, Kiessling F, Hennink WE, Storm G (2012). Drug targeting to tumors: principles, pitfalls and (pre-) clinical progress. J. Control Release.

[10] Manaia EB (2017). Physicochemical characterization of drug nanocarriers. Int. J. Nanomed..

[11] Etheridge ML (2013). The big picture on nanomedicine: the state of investigational and approved nanomedicine products. Nanomedicine.

[12] Bobo D, Robinson KJ, Islam J, Thurecht KJ, Corrie SR (2016). Nanoparticle-based medicines: a review of fda-approved materials and clinical trials to date. Pharm. Res..

[13] Rigler, R. & Elson, E. *Fluorescence Correlation Spectroscopy: Theory and Applications*. (Springer-Verlag Berlin Heidelberg, 2001).

[14] Hess ST, Huang S, Heikal AA, Webb WW (2002). Biological and chemical applications of fluorescence correlation spectroscopy: a review. Biochemistry.

[15] Kim SA, Schwille P (2003). Intracellular applications of fluorescence correlation spectroscopy: prospects for neuroscience. Curr. Opin. Neurobiol..

[16] Koynov K, Butt HJ (2012). Fluorescence correlation spectroscopy in colloid and interface science. Curr. Opin. Colloid Interface Sci..

[17] Mukhopadhyay A, Zhao J, Bae SC, Granick S (2002). Contrasting friction and diffusion in molecularly thin confined films. Phys. Rev. Lett..

[18] Papadakis CM, Košovan P, Richtering W, Wöll D (2014). Polymers in focus: fluorescence correlation spectroscopy. Colloid Polym. Sci..

[19] Woll D (2014). Fluorescence correlation spectroscopy in polymer science. RSC Adv..

[20] Zhao J, Granick S (2004). Polymer lateral diffusion at the solid–liquid interface. J. Am. Chem. Soc..

[21] Hemmelmann M, Kurzbach D, Koynov K, Hinderberger D, Zentel R (2012). Aggregation behavior of amphiphilic p(HPMA)-co-p(LMA) copolymers studied by FCS and EPR spectroscopy. Biomacromolecules.

[22] Weyermann J (2004). Physicochemical characterisation of cationic polybutylcyanoacrylat-nanoparticles by fluorescence correlation spectroscopy. Eur. J. Pharm. Biopharm..

[23] Nuhn L (2012). Cationic nanohydrogel particles as potential siRNA carriers for cellular delivery. ACS Nano.

[24] Rigler P, Meier W (2006). Encapsulation of fluorescent molecules by functionalized polymeric nanocontainers: investigation by confocal fluorescence imaging and fluorescence correlation spectroscopy. J. Am. Chem. Soc..

[25] Fritz T (2016). Orthogonal click conjugation to the liposomal surface reveals the stability of the lipid anchorage as crucial for targeting. Chemistry.

[26] Dakwar GR (2014). Colloidal stability of nano-sized particles in the peritoneal fluid: Towards optimizing drug delivery systems for intraperitoneal therapy. Acta Biomater..

[27] Novo L (2015). Targeted decationized polyplexes for siRNA delivery. Mol. Pharm..

[28] Milani S, Baldelli Bombelli F, Pitek AS, Dawson KA, Rädler J (2012). Reversible versus irreversible binding of transferrin to polystyrene nanoparticles: soft and hard corona. ACS Nano.

[29] Kohli I, Alam S, Patel B, Mukhopadhyay A (2013). Interaction and diffusion of gold nanoparticles in bovine serum albumin solutions. Appl. Phys. Lett..

[30] Pelaz B (2015). Surface functionalization of nanoparticles with polyethylene glycol: effects on protein adsorption and cellular uptake. ACS Nano.

[31] Maffre P, Nienhaus K, Amin F, Parak WJ, Nienhaus GU (2011). Characterization of protein adsorption onto FePt nanoparticles using dual-focus fluorescence correlation spectroscopy. Beilstein J. Nanotechnol..

[32] Alam S, Mukhopadhyay A (2014). Conjugation of gold nanorods with bovine serum albumin protein. J. Phys. Chem. C..

[33] Nuhn L (2014). Degradable cationic nanohydrogel particles for stimuli-responsive release of siRNA. Macromol. Rapid Commun..

[34] Mittag JJ (2017). Impact of plasma protein binding on cargo release by thermosensitive liposomes probed by fluorescence correlation spectroscopy. Eur. J. Pharm. Biopharm..

[35] Kapusta, P. Absolute diffusion coefficients: compilation of reference data for FCS calibration, Application Note (PicoQuant GmbH, Berlin), http://www.picoquant.com/technotes/appnote_diffusion_coefficients.pdf (2010).

[36] Hörtz C (2015). Cylindrical brush polymers with polysarcosine side chains: a novel biocompatible carrier for biomedical applications. Macromolecules.

[37] Gösch M, Blom H, Holm J, Heino T, Rigler R (2000). Hydrodynamic flow profiling in microchannel structures by single molecule fluorescence correlation spectroscopy. Anal. Chem..

[38] Wennmalm S, Thyberg P, Xu L, Widengren J (2009). Inverse-fluorescence correlation spectroscopy. Anal. Chem..

[39] Armstrong JK, Wenby RB, Meiselman HJ, Fisher TC (2004). The hydrodynamic radii of macromolecules and their effect on red blood cell aggregation. Biophys. J..

[40] Williams RJ, Lipowska M, Patonay G, Strekowski L (1993). Comparison of covalent and noncovalent labeling with near-infrared dyes for the high-performance liquid chromatographic determination of human serum albumin. Anal. Chem..

[41] Berezin MY, Lee H, Akers W, Nikiforovich G, Achilefu S (2007). Ratiometric analysis of fluorescence lifetime for probing binding sites in albumin with near-infrared fluorescent molecular probes. Photochem. Photobiol..

[42] Berezin MY (2011). Rational approach to select small peptide molecular probes labeled with fluorescent cyanine dyes for in vivo optical imaging. Biochemistry.

[43] Klinker K (2017). Secondary-structure-driven self-assembly of reactive polypept(o)ides: controlling size, shape, and function of core cross-linked nanostructures. Angew. Chem. Int. Ed..

[44] Schäfer O (2017). Combining orthogonal reactive groups in block copolymers for functional nanoparticle synthesis in a single step. ACS Macro Lett..

[45] Rijcken CJF, Talelli M, van Nostrum CF, Storm G, Hennink WE (2010). Therapeutic Nanomedicine: cross linked micelles with transiently linked drugs – a versatile drug delivery system. Eur. J. Nanomed..

[46] Talelli M (2015). Core-crosslinked polymeric micelles: principles, preparation, biomedical applications and clinical translation. Nano Today.

[47] Rijcken, C. J. F. Tuneable and Degradable Polymeric Micelles for Drug Delivery: from Synthesis to Feasibility In Vivo. (Utrecht University, 2007).

[48] Heissig P, Schrimpf W, Hadwiger P, Wagner E, Lamb DC (2017). Monitoring integrity and localization of modified single-stranded RNA oligonucleotides using ultrasensitive fluorescence methods. PLoS One.

[49] Geary RS (2009). Antisense oligonucleotide pharmacokinetics and metabolism. Expert Opin. Drug. Metab. Toxicol..

[50] Shang L, Nienhaus GU (2017). In situ characterization of protein adsorption onto nanoparticles by fluorescence correlation spectroscopy. Acc. Chem. Res..

[51] Wang H (2016). The nature of a hard protein corona forming on quantum dots exposed to human blood serum. Small.

[52] Carril M (2017). In situ detection of the protein corona in complex environments. Nat. Commun..

[53] Zou Q (2017). Biological photothermal nanodots based on self-assembly of peptide–porphyrin conjugates for antitumor therapy. J. Am. Chem. Soc..

[54] Fu M, Wang A, Zhang X, Dai L, Li J (2015). Direct observation of the distribution of gelatin in calcium carbonate crystals by super-resolution fluorescence microscopy. Angew. Chem. Int. Ed..

