# Supplementary material for: Monitoring drug nanocarriers in human blood by near-infrared fluorescence correlation spectroscopy
Source: Nat Commun. 2018 Dec 13;9:5306. doi: 10.1038/s41467-018-07755-0 (PMC6294246; doi:10.1038/s41467-018-07755-0)
Supplement: Supplementary file 1 — Supplementary Information [file 41467_2018_7755_MOESM1_ESM.pdf]

Supplementary Information

Monitoring Drug Nanocarriers in Human Blood by Near-Infrared Fluorescence Correlation Spectroscopy

Inka Negwer[1,2], Andreas Best[1], Meike Schinnerer[3], Olga Schäfer[4], Leon Capeloa[4], Manfred Wagner[1], Manfred Schmidt[3], Volker Mailänder[5,1], Mark Helm[2], Matthias Barz[4], Hans-Jürgen Butt[1,6,*] and Kaloian Koynov[1,*]

[1]*Max Planck Institute for Polymer Research, Ackermannweg 10, 55128 Mainz (Germany)*

[2]*Pharmaceutical Chemistry, Institute of Pharmacy and Biochemistry, Johannes Gutenberg University, Staudinger Weg 5, 55128 Mainz (Germany)*

[3]*Institute of Physical Chemistry, Johannes Gutenberg University, Jakob Welder Weg 11, 55128 Mainz (Germany)*

[4]*Institute of Organic Chemistry, Johannes Gutenberg University, Duesbergweg 10-14, 55128 Mainz (Germany)*

[5]*Department of Dermatology, University Medical Center of the Johannes Gutenberg University, Langenbeckstr. 1, 55131 Mainz (Germany)*

[6]*Earth-Life Science Institute, Tokyo Institute of Technology, Meguro, Tokyo 152-8551 (Japan)*

[*]corresponding authors: koynov@mpip-mainz.mpg.de, butt@mpip-mainz.mpg.de

Supplementary Notes

Note 1: NIR-FCS measurements in static blood

In order to correlate the recorded FCS curves with the precise position of the observation volume V_{obs} with respect to the blood cells, the cells were stained with the green-fluorescing membrane dye DiI (see Supplementary Method). During the staining procedure, plasma was removed and substituted by phosphate buffered saline (PBS) to which **CB1** was added in a nanomolar concentration. In this blood cells suspension (BCS), images recorded with the LSM 880 confocal microscope rendered a first impression of the 3D configuration of the cells. However, in the visible spectrum the main components of red blood cells, oxy- and deoxyhemoglobin, show strong absorption leading to a dramatic loss in signal intensity already at the first micrometers.[1] Strong scattering additionally reduced resolution with the effect that a clear distinction of cellular structures at imaging depths >10 µm was not possible. Within the first 10 µm we observed a close network of packed, sedimented cells (Supplementary Fig. 4). FCS measurements were performed at penetration depths between 0 and 10 µm (Supplementary Fig. 4a). When V_{obs} was positioned in a cell free spot clear off the glass-sample interface, the corresponding autocorrelation curve could be fitted with a fit function for one type of freely diffusing species (eq.3 with m=1 in the main text) (Supplementary Fig. 4b). The diffusion coefficient value 21.5 µm² s^{-1} obtained from the fit differed only marginally from that in pure water, $D_{\mathbf{CB1}, \text{water}}$ = 20.4 µm² s^{-1} assuming that the dimensions of V_{obs} were equal in both cases. However, when the measurement was performed in a spot partially occupied by a cell, the detected fluorescence intensity and the amplitude of the autocorrelation curve decreased significantly. Furthermore, the fits to the autocorrelation curve did not yield reliable values for D (Supplementary Fig. 4c). We concluded that because free Brownian diffusion of the fluorescent tracers can occur only in the liquid fraction of the blood, the full or partial obstruction of the FCS observation volume by cells prevented reliable measurements.

FCS is sensitive to spherical aberrations caused by a mismatch of refractive indices. Here we used a water immersion microscope objective that is optimized for water with n_{water} = 1.33.[2] Red blood cells on the other hand were reported to have a refractive index of 1.399.[3] Thus, passing the laser beam through a cell might lead to an increase in V_{obs} resulting in longer diffusion times and correspondingly a lower diffusion coefficient. We therefore investigated if a measurement at locations above the first layer of cells yielded comparable results to those where no cells crossed the laser beam (Supplementary Fig. 4d-i). By positioning V_{obs} at such a spot a good autocorrelation curve was measured (Supplementary Fig. 4h). The fit to this curve yielded a diffusion coefficient of 19.3 µm² s^{-1} that is only slightly lower than $D_{\mathbf{CB1}, \text{water}}$. Thus, we conclude that laser penetration through one layer of cells did not distort the observation volume.

Next, we sought to measure the diffusion in full blood by FCS (Supplementary Fig. 5). Here, staining of the cell membrane was omitted to preserve the original blood composition. This, however, meant that we could not control if the spot was occupied by a cell or not. Subsequently, we searched for spots that showed sufficient fluorescence signal intensity. In some cases, a slow movement of cells on a second to minutes time scale could be observed by fluctuations of the average count rate. Furthermore, we assessed that at penetration depths of >10 µm the likelihood of finding good spots decreased. We assumed that this was caused by the increasing distortion of V_{obs} when multiple layers of cells were passed by the laser beam. The FCS measurements were therefore performed at a depth of penetration of 10 µm and at 10 different lateral positions in the sample. The average value of the diffusion coefficient obtained in this way was $D_{\mathbf{CB1},\ \mathrm{stat.\ blood}}$ = 10.0 ± 2.0 µm² s^{-1}. Due to undefined position of the FCS observation volume, this value should be considered only as a rough approximation.

Note 2: NIR-FCS studies in plasma

The influence of the liquid blood fraction was evaluated by studying **CB1** and **CB2** diffusion in undiluted human plasma. The diffusion time of **CB1** in plasma increased by a factor of 1.43 compared to that measured in water (Supplementary Fig. 6). This could be the consequence of several effects that require careful consideration.

(1) As plasma contains proteins, ions, and other solutes its refractive index may be slightly higher than that of water. This effect combined with an enhanced scattering in plasma might lead to a slight increase of the FCS observation volume in plasma compared to water especially at higher penetration depths. To address this issue we recorded FCS autocorrelation curves at different penetration depths in water and plasma (Supplementary Fig. 6b). The results showed no change in the diffusion time of **CB1** for penetration depths up to 50 µm in plasma. Thus, we conclude that up to this penetration depth the observation volume V_{obs} remains unaffected and used its radial dimension r_0 calibrated in water to calculate the diffusion coefficient of **CB1** in plasma, $D_{\mathbf{CB1},\ \mathrm{plasma}}$ of 14.3 µm² s^{-1}.

(2) Many nanocarriers are reported to show an immediate adsorption of plasma proteins upon contact with plasma or blood. In 2007, Cedervall *et al*. coined the term "protein corona" to describe this behavior.[4] The eventual presence of such tightly bound layer of strongly adsorbed proteins would increase the hydrodynamic radius of **CB1** and thus its diffusion time in plasma compared to water. Using Stokes–Einstein relation and assuming the viscosity of water for the plasma, the diffusion coefficient of **CB1** in plasma $D_{\mathbf{CB1},\ \mathrm{plasma}}$=14.3 µm²s^{-1} translates to a hydrodynamic radius of 16.2 nm. That is an increase with ΔR_{H} = 4.8 nm as compared to the value in water. In order to get further insight, we also studied the second, larger cylindrical polymer brush, **CB2**. The diffusion coefficient of **CB2** in water was determined by dynamic light scattering ($D_{\mathbf{CB2},\mathrm{water}}$ = 10.9 µm² s^{-1}, $R_{H,\mathbf{CB2}}$ = 21.3 nm). In

plasma (Supplementary Fig. 7), we found again a reduction of the diffusion coefficient compared to water by a factor of 1.43 ($D_{\mathbf{CB2},\text{plasma}}$ = 7.6 µm² s^{-1}), that translates into a ΔR_H of 9.4 nm again assuming water viscosity for plasma in the Stokes –Einstein (eq. 5 in the main text) relation. Considering the analogous synthetic identity of both cylindrical polymer brushes a protein adsorption layer would be expected to consist of the same proteins and therefore give a similar ΔR_H. However, as the two differently sized cylindrical polymer brushes showed an identical scaling factor between diffusion in water and plasma and thus different ΔR_H we deduced that the difference could not be explained by the formation of a tightly bound protein corona.

(3) Due to its high protein content, plasma has a higher macroscopic viscosity than water. Rolling ball viscometer measurements yielded η_{plasma} = 1.48 mPa*s at 22°C and 1.35 mPa*s at 25°C. Clearly, these values for the macroscopic viscosity of the plasma have to be used with care when the diffusion of small species such as **CB1** in a complex environment as the blood plasma is considered and their diffusion coefficient calculated through the Stokes-Einstein relation (eq.5). On one hand, fluorescence recovery after photobleaching studies[5] have shown that the concentration dependence of the long-time self-diffusion coefficient of hard-sphere colloids is very similar to the concentration dependence of the macroscopic viscosity at low volume fractions. On the other hand, FCS studies of tracer diffusion in various crowded enviroments[6-8] have shown that the local viscosity or friction that is experienced by the tracers depends on their size as well as on the characteristic length scale of the surrounding matrix and does not necessarily equal the macroscopic viscosity. Nevertheless, the fact that both **CB1** and **CB2** show the same diffusion slowdown of 1.43 in plasma compared to water strongly suggest that in plasma both cylindrical polymer brushes do not change their size but simply experience an effective viscosity that is 1.43-times higher than that of water. It is worth to note that this slowdown in the **CB1** and **CB2** tracer diffusion coefficient in plasma compared to water, closely matches the value of 1.4 that was reported[9] for the slowdown in the self-diffusion coefficient of human serum albumin and four other proteins in solutions with high ionic strength and a protein volume fraction of 6%.

Note 3: Combining normal and inverse FCS

A precise analytical derivation of eq. 2 in the main text that combines normal and inverse FCS is outside of the scope of this paper. As discussed below such combination is justified by the order of magnitude difference in the sizes and thus in the diffusion times of the fluorescent species and the blood cells. Indeed, the analytical expression for inverse-FCS (that is used as a part of eq. 2 in the main text) was initially derived by Wennmalm et al.[10] for strongly and homogeneously fluorescent media surrounding unlabeled nanoparticles with sizes in the range of 100nm. The medium was in fact a concentrated solution of small dye molecules. The idea is that, due to the high concentration of the

dye molecules their diffusion in and out of the FCS observation volume will not create intensity fluctuations. On the other hand while diffusing through the observation volume the unlabeled nanoparticles replace some of the fluorescent molecules, which leads to fluctuations (drops) in the measured fluorescent intensity. The obtained autocorrelation curve is then fitted with a single component to obtain the diffusion time of the unlabeled nanoparticles (in the order of 10 ms) and thus their diffusion coefficient.

In our case, the unlabeled objects are the red blood cells that are much larger than the nanoparticles used by Wennmalm et al.[10]. Thus, the diffusion time of the blood cells through the observation volume is very slow ~ 500 ms. In the time intervals, in which the observation volume is not occupied by blood cells, it is occupied by the studied fluorescent species (small dye molecules or loaded nanocarriers). In these time intervals high fluorescence intensity is measured. Clearly, this intensity is not constant as in the initial work of Wennmalm et al.[10], because due to the relatively low concentration of the studied fluorescent species their diffusion in and out of the observation volume creates intensity fluctuations. However, these fluctuations, caused by small species (~10 nm) with diffusion times in the order of a few ms are orders of magnitude faster than the ones caused by the blood cells. This very large difference in the time scales justifies the use of eq. 2 (in the main text) with two components: one for the small fluorescent species and one for the very large blood cells.

Supplementary Methods

Diffusion coefficient measurements by PFG NMR

The diffusion coefficient of IRDye®800CW-DBCO in aqueous solutions was measured using a 5 mm triple resonance TXI $^{1}H/^{13}C/^{15}N$ probe equipped with a z-gradient on the 850 MHz Bruker AVANCE III system. The temperature was kept at 25°C and regulated by a standard ^{1}H methanol NMR sample using the "topspin 3.1" software (Bruker). The control of the temperature was realized with a VTU (variable temperature unit) and an accuracy of +/- 0.1°C. The gradient strength was varied in 32 steps from 2 % to 100 %. The diffusion time was optimised at 50 ms and the gradient length to 1.4 ms. The relaxation delay was set to 3s. The gradient strength was calibrated by analysis of a sample of $^{2}H_2O/^{1}H_2O$ at a defined temperature and comparison with the theoretical diffusion coefficient of $^{2}H_2O/^{1}H_2O$ (values taken from Bruker diffusion manual).

For the diffusion measurements a 2D sequence (DOSY, stebpgp1s19) with a stimulated echo was used additionally with water suppression (3-9-19 pulse sequence with gradients).[11] The 2D NMR sequences for measuring diffusion coefficient uses echoes for convection compensation and longitudinal eddy current delays to store the magnetization in the z-axis, and only be dependent on T_1-relaxation. The calculation of the diffusion value was automatically done with the mono exponential function.[12]

Diffusion gradient amplitudes were varied linearly from 1 to 53 G/cm (10 to 470 mT/m) over a total of 32 experiments to achieve a strong diffusion weighting. Diffusion coefficients were then calculated for the integrated peak areas using an exponential decay fit function over the 16 spectra

$$S_i = S_0 \cdot \exp(-D \cdot b_i) \quad (1)$$

where the diffusion sensitivity factor *b* was calculated as given in the sequence description:

$$b_i = (2\pi \cdot \gamma \cdot G_i \cdot \delta)^2 \cdot \left(\Delta - \frac{\delta}{3}\right) \quad (2)$$

Typical spectra measured for aqueous solution of the IRDye®800CW-DBCO are shown in the Supplementary Figure 3. The fit yielded a value of 265 ± 10 µm² s^{-1} for the diffusion coefficient of the dye at 25°C that corresponds to a value of 251 ± 10 µm² s^{-1} at 23°C.

Staining of red blood cell membrane

Human blood (excess from a buffy coat preparation in our institute) was diluted with phosphate buffered saline (D-PBS, Thermo Fisher Scientific, Waltham, MA, USA) and centrifuged at 3,000 g for 3 min. The supernatant was carefully removed, including a viscous layer of leukocytes, and the

remaining red blood cells were washed three times with 10 mL of D-PBS. After the last washing step, 50 µL of packed red blood cells were dispersed in 4 mL D-PBS and 1 mL of 5% glucose solution (anhydrous D(+)-glucose Carl Roth, Karlsruhe, Germany). To this, 50 µL of DiI (0.5 mg mL^{-1}, Molecular Probes, Life technologies, Eugene, OR, USA) were added and the mixture was incubated at room temperature for 30 min. Next, the red blood cells were sedimented by centrifugation as described above and washed three times with D-PBS. At the last step, 100 µL of D-PBS were added to the packed red blood cells resulting in a hematocrit of ~ 30%.

For confocal imaging on the LSM 880 microscope, DiI was excited at 543 nm and the emission was detected with the integrated Quasar detector in the range from 553 to 633 nm. The pinhole was set to 38 µm. The average pixel dwell time was 1.5 µs and image resolution was 512 x 512 pixels.

Supplementary Figures

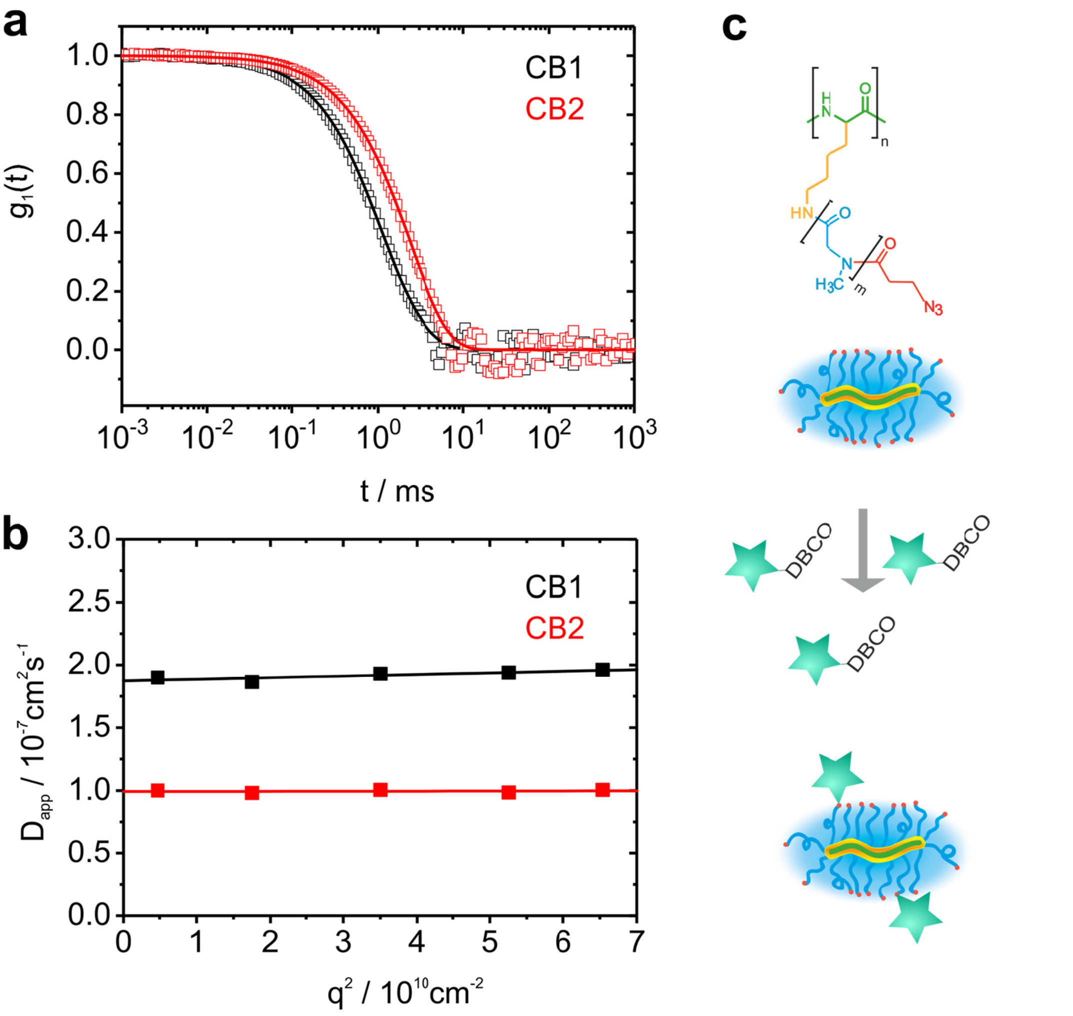

Supplementary Figure 1: Multi-angle DLS measurements of the cylindrical polymer brushes **CB1** and **CB2** in diluted aqueous solutions (PBS) at 20°C. a) Autocorrelation functions at 30° scattering angle for **CB1** (black squares) and **CB2** (red squares). b) Apparent diffusion coefficient as function of q^2 for **CB1** (black squares) and **CB2** (red squares). Using the temperature dependence of the water viscosity and the Stokes-Einstein relation (eq. 5 in the main text) we calculated the values of the diffusion coefficients of **CB1** and **CB2** in aqueous solutions at 23°C as $D_{\mathbf{CB1},\text{ water}}$ = 20.4 µm² s^{-1} and that of **CB2** as $D_{\mathbf{CB2},\text{ water}}$ = 10.9 µm² s^{-1}. c) Schematic structure of **CB1** and labeling with IRDye®800CW-DBCO by strain-promoted azide-alkyne cycloaddition.

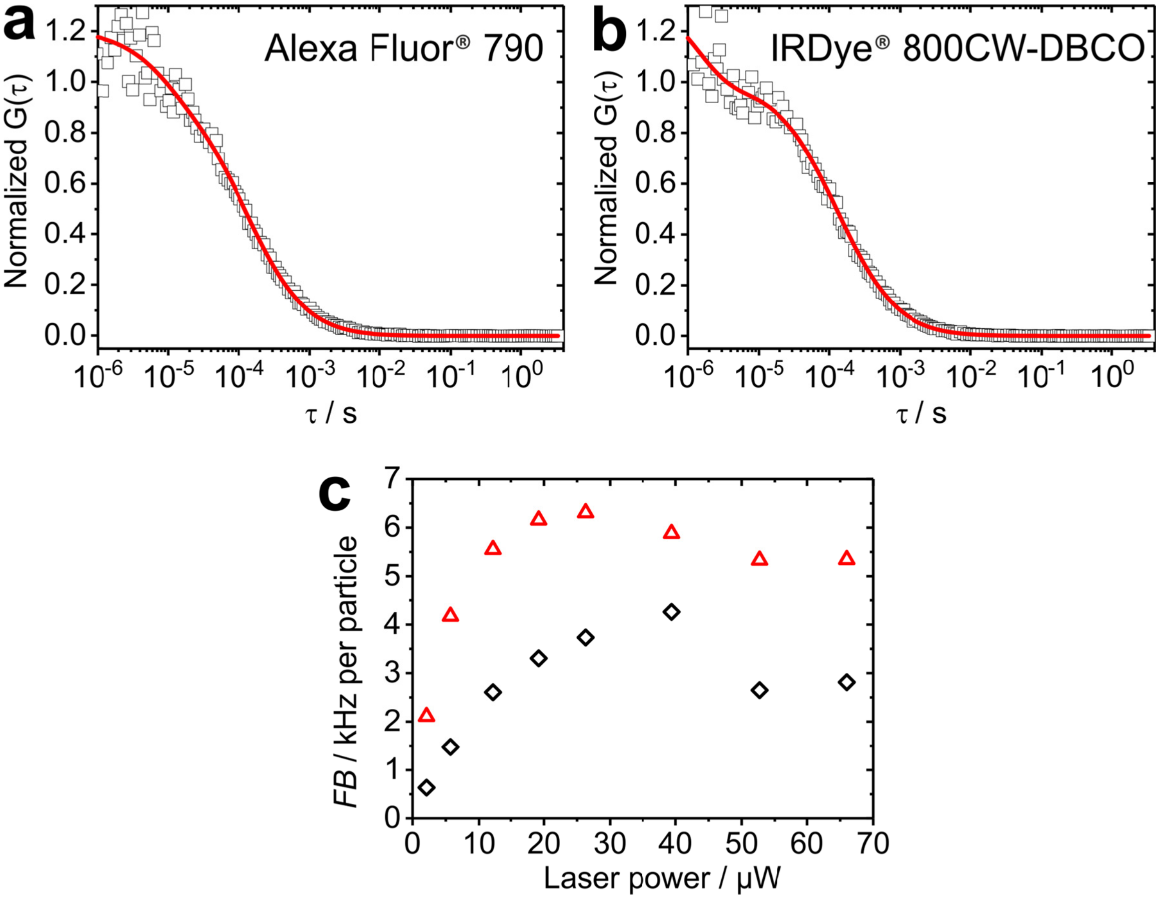

Supplementary Figure 2: Normalized FCS autocorrelation curves (black squares) of a) Alexa Fluor®790 and b) IRDye®800CW-DBCO measured in aqueous solutions at 23°C. The solid red lines represent the corresponding single component fits (eq. 3 with *m*=1 in the main text) yielding the diffusion times of the dyes. c) Fluorescence brightness *FB* of IRDye®800CW-DBCO (black diamonds) and the labeled cylindrical polymer brush **CB1** (red triangles) vs. excitation laser power. The power values were measured in air after the objective. In all other NIR-FCS measurements, laser power was limited to 6 μW to avoid saturation.

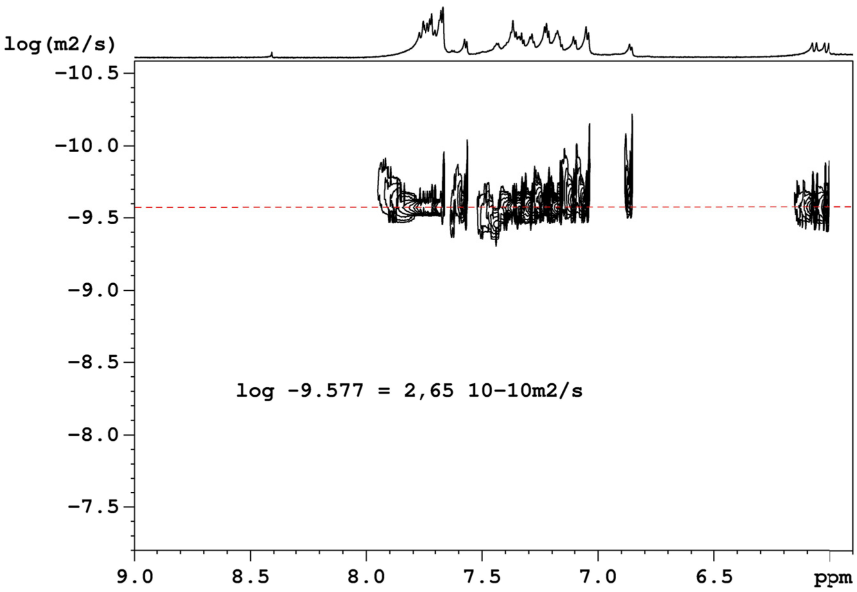

Supplementary Figure 3: NMR DOSY measurements of IRDye®800CW-DBCO were done in water at 850 MHz and 25°C. The y-axis represents the log of the diffusion coefficient values and the x-axis represents the ^{1}H-ppm scale. The red dashed line corresponds to a mono exponential fit that yields the diffusion coefficient. Using the temperature dependence of the water viscosity and the Stokes-Einstein relation (eq. 5 in the main text) we calculated the value of the diffusion coefficients of IRDye®800CW-DBCO in water at 23°C as $D_{\mathbf{IRDye®800CW\text{-}DBCO,\ water}}$ = 251 ± 10 µm² s^{-1}.

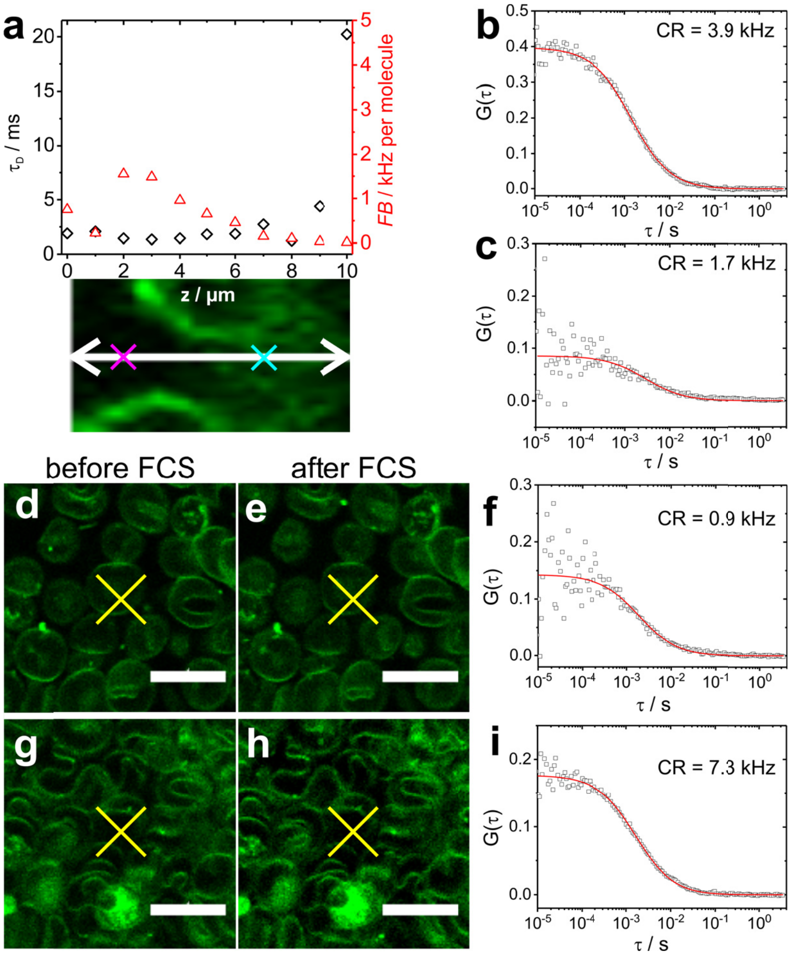

Supplementary Figure 4: NIR-FCS measurements of **CB1** in stained blood cell suspension (BCS). a) Diffusion time (τ_D) and fluorescence brightness (FB) for FCS measurements of **CB1** performed at *z* positions (penetration depth) ranging from 0 to 10 µm. The *x,z*-confocal image below the graph depicts whether the corresponding *z* position was occupied by a cell or not. b) Autocorrelation curve recorded at a cell free position (pink crosshairs in a)) at 2 µm penetration depth. Inserted in the graph is the average fluorescence intensity (count rate, CR). The fitting yields $D_{\mathbf{CB1},\,\mathrm{BCS}}$ = 21.5 µm² s^{-1}. c) Autocorrelation curve measured at an occupied by cells spot (blue crosshairs in a)) at 7 µm penetration depth. CR is decreased and fitting yields $D_{\mathbf{CB1},\,\mathrm{BCS}}$ = 11.4 µm² s^{-1}. Measurements in cell occupied positions (d-f) at 1 µm penetration depth, and above the cell (g-i) at 4 µm penetration depth. d) and g) show the position of the V_{obs} before the FCS measurement. e) and h) show the position of V_{obs} after the FCS measurement and verify that no substantial movement of the cells occurred during the experiment. f) The corresponding autocorrelation curve shows a low CR and yields $D_{\mathbf{CB1},\,\mathrm{BCS}}$ = 16.6 µm² s^{-1}. i) Autocorrelation curve above one layer of cells yields $D_{\mathbf{CB1},\,\mathrm{BCS}}$ = 19.3 µm² s^{-1} at a high CR. Fitting (eq. 3 with m=1 and f_T =0 in the main text) was performed from a lag time of 10 µs and did not include a triplet contribution. All diffusion coefficients were calculated assuming that the V_{obs} does not depend on the measurement position (identical dimensions r_0, z_0). Scale bar: 10 µm.

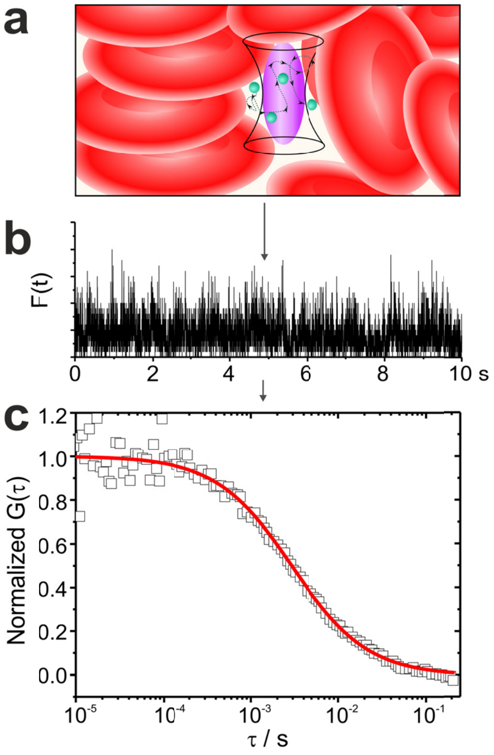

Supplementary Figure 5: NIR-FCS measurements of **CB1** in static human heparin-treated blood. a) In the crowded environment of blood cells, V_{obs} has to be positioned in a cell free spot in order to monitor diffusion. b) Excerpt of the fluorescence intensity time trace in a cell free position of V_{obs}. c) The experimental autocorrelation curve (symbols) and the corresponding single component fit (solid line) performed from a lag time of 10 µs and without triplet contribution (eq. 3 with m=1 and f_T =0 in the main text). The fitting yielded $D_{\mathbf{CB1}, \text{static blood}}$ = 10.2 µm² s^{-1} assuming that the V_{obs} does not depend on the measurement position (identical dimensions r_0, z_0).

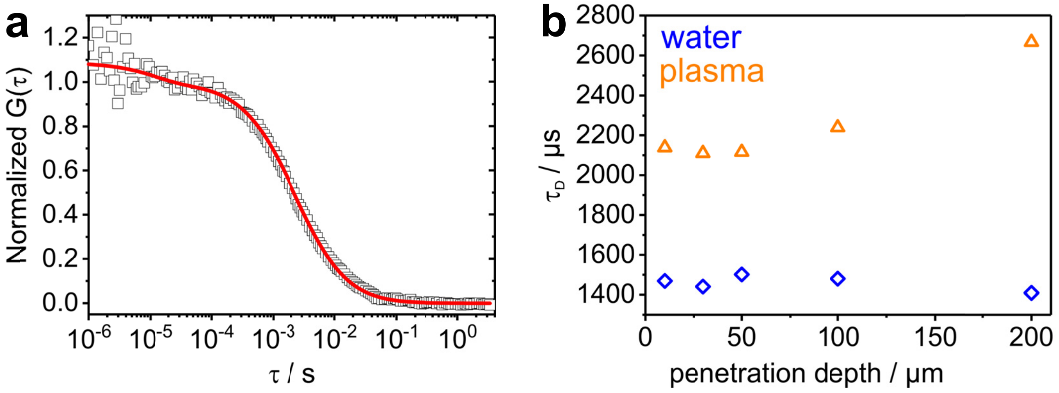

Supplementary Figure 6: a) Normalized NIR-FCS autocorrelation curve (black squares) measured for **CB1** in human citrate plasma at penetration depth of 30 µm and (red line) the corresponding one component fit (eq. 3 with *m*=1 in the main text). b) Diffusion time τ_D of **CB1**, obtained from the fits to the autocorrelation curves measured at different penetration depths in plasma (orange triangles) and water (blue diamonds). In plasma, τ_D increased at penetration depths above 50 µm presumably due to the effect of a refractive index mismatch that leads to an increase in the FCS observation volume. The τ_D measured at penetration depth of 30 µm was used to calculate the diffusion coefficient of **CB1** in plasma $D_{\mathbf{CB1},\ \mathrm{plasma}}$ = 14.3 µm^2 s^{-1}.

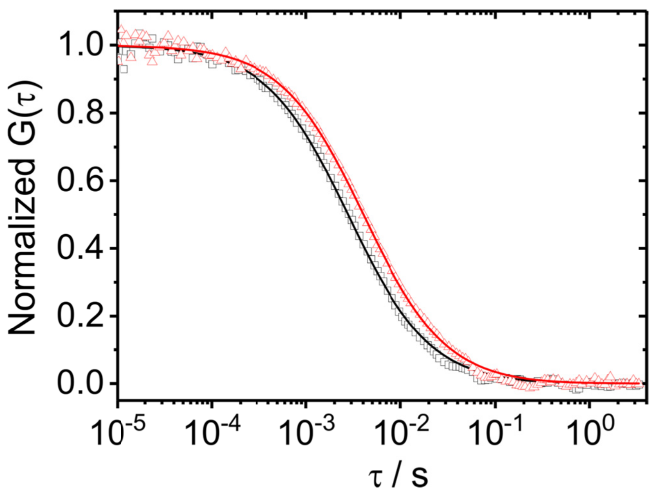

Supplementary Figure 7: Normalized NIR-FCS autocorrelation curve (red triangles) measured for **CB2** in human citrate plasma at penetration depth of 30 µm. Fitting (red line) was performed with a one component fit (eq. 3 with *m*=1 in the main text) and yielded the diffusion time and consequently the diffusion coefficient of **CB2** in plasma $D_{\mathbf{CB2},\ \mathrm{plasma}}$ = 7.6 µm² s^{-1}. The corresponding data for **CB2** in water (black) are also shown for comparison.

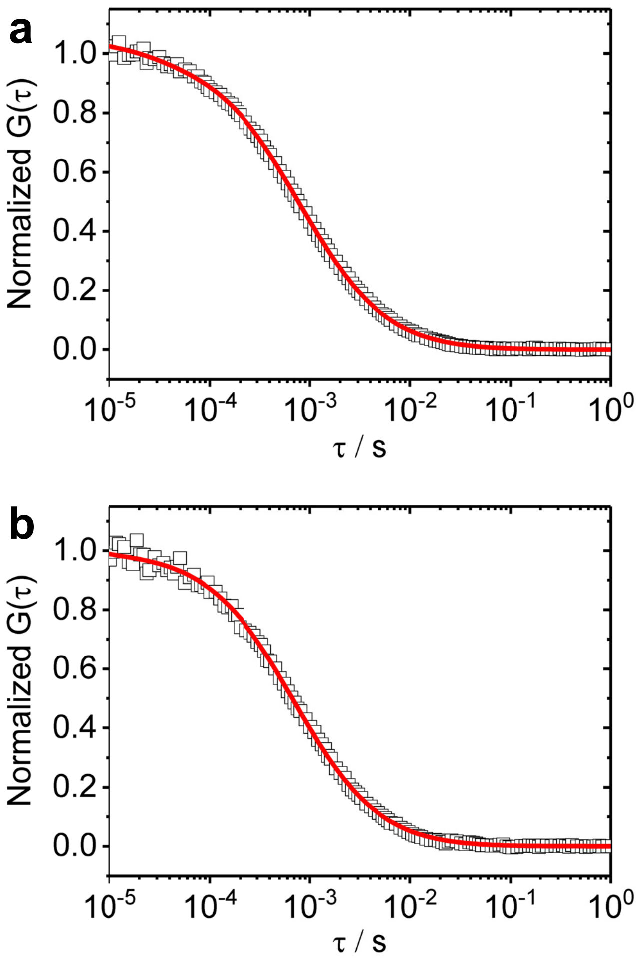

Supplementary Figure 8: Normalized autocorrelation curves (black squares) measured for IRDye®800CW-DBCO in (a) human citrate plasma and (b) PBS solution (50 mg/ml) of HSA. Fitting (red lines) was performed with a one component fit (eq. 3 with *m*=1 in the main text) and yielded $D_{\text{IRDye®800CW-DBCO, plasma}}$ = 38.1 $\mu m^2\ s^{-1}$ and $D_{\text{IRDye®800CW-DBCO, HSA}}$ = 47.7 $\mu m^2\ s^{-1}$. After accounting for the viscosities of the corresponding solutions these values translate to hydrodynamic radii $R_{\text{H IRDye®800CW-DBCO, plasma}}$ = 4.3 nm and $R_{\text{H IRDye®800CW-DBCO, HSA}}$ = 4.0 nm.

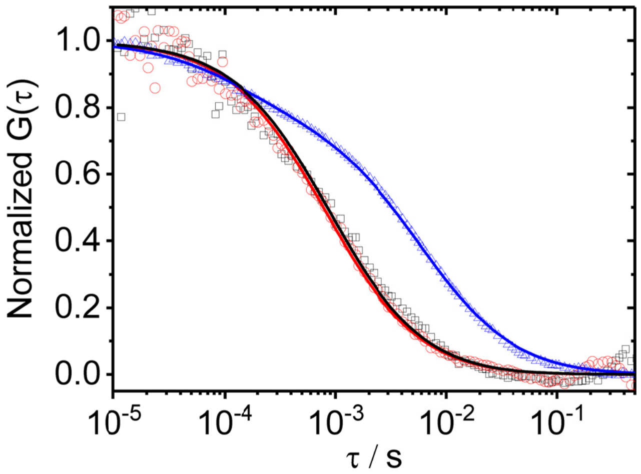

Supplementary Figure 9: NIR-FCS studies of the leakage kinetic for IRDye®800CW non-covalently loaded in core-crosslinked micelle nanocarriers **M2** in human blood. Normalized autocorrelation curve (blue triangles) measured for **M2** in water and (blue line) the corresponding two component fit (eq.3 with *m*=2 in the main text). Normalized autocorrelation curves for **M2** in blood measured after incubation time of 30 minutes (black squares) and 30 hours (red circles) and subtracting the cell contribution (eq. 6 in the main text). The black and the red solid lines represent the corresponding single component fits (eq.1 with *m*=1 in the main text). The results show that while in water the dye is mainly loaded in **M2**, in blood it is fully released already after 30 min.

Supplementary Tables:

Supplementary Table 1: Characterization of cylindrical polymer brushes **CB1** and **CB2**.

Sample	$\langle \frac{1}{R_h} \rangle_z^{-1}$ / nm[a)]	Mw / g·mol^{-1} [b)]	R_g / nm[c)]	ζ / mV[d)]	Aspect ratio (AFM)[e)]
**CB1**	11 $\pm$ 1	2.2E5 $\pm$ 0.1E5	10.4 $\pm$ 1.0	-6 $\pm$ 4	1.6
**CB2**	21 $\pm$ 1	10.4E5 $\pm$ 0.5E5	22.7 $\pm$ 2.3	-3 $\pm$ 5	3.3

a) determined by multi-angle dynamic light scattering in PBS (20°C)

b, c) determined by static light scattering in PBS

d) determined by zeta potential measurements in 10 mM NaCl

e) determined by atomic force microscopy on mica substrates

Supplementary Table 2: Characterization of unlabeled core-crosslinked micelles **M**.

Sample	$\langle \frac{1}{R_h} \rangle_z^{-1}$ / nm[a)]	ζ / mV[b)]
**M**	45$\pm$ 1	-1 ±4

a) determined by multi-angle dynamic light scattering in PBS (20°C)

b) determined by zeta potential measurements in 10 mM NaCl

Supplementary References

1 Altinoglu, E. I. & Adair, J. H. Near infrared imaging with nanoparticles. *Wiley Interdiscip. Rev. Nanomed. Nanobiotechnol.* **2**, 461-477 (2010).

2 Leroux, C. E., Wang, I., Derouard, J. & Delon, A. Adaptive optics for fluorescence correlation spectroscopy. *Opt. Express* **19**, 26839-26849 (2011).

3 Park, Y. *et al.* Refractive index maps and membrane dynamics of human red blood cells parasitized by Plasmodium falciparum. *Proc. Natl. Acad. Sci. U.S.A.* **105**, 13730-13735 (2008).

4 Cedervall, T. *et al.* Understanding the nanoparticle-protein corona using methods to quantify exchange rates and affinities of proteins for nanoparticles. *Proc. Natl. Acad. Sci. U.S.A.* **104**, 2050-2055 (2007).

5 van Blaaderen, A., Peetermans, J., Maret, G. & Dhont, J. K. G. Long - time self - diffusion of spherical colloidal particles measured with fluorescence recovery after photobleaching. *J. Chem. Phys.* **96**, 4591-4603 (1992).

6 Szymanski, J., Patkowski, A., Wilk, A., Garstecki, P. & Holyst, R. Diffusion and viscosity in a crowded environment: from nano- to macroscale. *J. Phys. Chem. B* **110** (2006).

7 Cherdhirankorn, T. *et al.* Diffusion in polymer solutions studied by fluorescence correlation spectroscopy. *J. Phys. Chem. B* **113**, 3355-3359, doi:10.1021/jp809707y (2009).

8 Kalwarczyk, T. *et al.* Comparative analysis of viscosity of complex liquids and cytoplasm of mammalian cells at the nanoscale. *Nano Lett.* **11**, 2157-2163 (2011).

9 Le Bon, C., Nicolai, T., Kuil, M. E. & Hollander, J. G. Self-Diffusion and Cooperative Diffusion of Globular Proteins in Solution. *J. Phys. Chem B.* **103**, 10294-10299 (1999).

10 Wennmalm, S., Thyberg, P., Xu, L. & Widengren, J. Inverse-Fluorescence Correlation Spectroscopy. *Anal. Chem.* **81**, 9209-9215 (2009).

11 Jerschow, A. & Muller, N. Suppression of convection artifacts in stimulated-echo diffusion experiments. Double-stimulated-echo experiments. *J. Magn. Reson.* **125**, 372-375 (1997).

12 Stejskal, E. O. & Tanner, J. E. Spin Diffusion Measurements: Spin Echoes in the Presence of a Time-Dependent Field Gradient. *J. Chem. Phys.* **42**, 288-292 (1965).
